# Clonal CD8^+^ T Cell Persistence and Variable Gene Usage Bias in a Human Transplanted Hand

**DOI:** 10.1371/journal.pone.0136235

**Published:** 2015-08-19

**Authors:** Joseph Y. Kim, Arumugam Balamurugan, Kodi Azari, Christian Hofmann, Hwee L. Ng, Elaine F. Reed, Suzanne McDiarmid, Otto O. Yang

**Affiliations:** 1 Division of Infectious Diseases, Department of Medicine, Geffen School of Medicine, University of California Los Angeles, Los Angeles, California, United States of America; 2 Department of Microbiology, Immunology, and Molecular Genetics, Geffen School of Medicine, University of California Los Angeles, Los Angeles, California, United States of America; 3 Department of Orthopaedic Surgery, Geffen School of Medicine, University of California Los Angeles, Los Angeles, California, United States of America; 4 Department of Pathology and Laboratory Medicine, Geffen School of Medicine, University of California Los Angeles, Los Angeles, California, United States of America; 5 UCLA Immunogenetics Center, Geffen School of Medicine, University of California Los Angeles, Los Angeles, California, United States of America; 6 Division of Gastroenterology, Department of Pediatrics, Geffen School of Medicine, University of California Los Angeles, Los Angeles, California, United States of America; UNIFESP Federal University of São Paulo, BRAZIL

## Abstract

Immune prophylaxis and treatment of transplanted tissue rejection act indiscriminately, risking serious infections and malignancies. Although animal data suggest that cellular immune responses causing rejection may be rather narrow and predictable based on genetic background, there are only limited data regarding the clonal breadth of anti-donor responses in humans after allogeneic organ transplantation. We evaluated the graft-infiltrating CD8^+^ T lymphocytes in skin punch biopsies of a transplanted hand over 178 days. Profiling of T cell receptor (TCR) variable gene usage and size distribution of the infiltrating cells revealed marked skewing of the TCR repertoire indicating oligoclonality, but relatively normal distributions in the blood. Although sampling limitation prevented complete assessment of the TCR repertoire, sequencing further identified 11 TCR clonal expansions that persisted through varying degrees of clinical rejection and immunosuppressive therapy. These 11 clones were limited to three TCR beta chain variable (BV) gene families. Overall, these data indicate significant oligoclonality and likely restricted BV gene usage of alloreactive CD8^+^ T lymphocytes, and suggest that changes in rejection status are more due to varying regulation of their activity or number rather than shifts in the clonal populations in the transplanted organ. Given that controlled animal models produce predictable BV usage in T lymphocytes mediating rejection, understanding the determinants of TCR gene usage associated with rejection in humans may have application in specifically targeted immunotherapy.

## Introduction

Solid organ transplantation is frequently the only option for patients with terminal organ failure, but poses a clinical management challenge in balancing the risk of rejection versus infection. The major advance that has made transplantation a viable strategy is the development of immunosuppressive drugs that both prevent and treat immune-mediated graft rejection, most of which act on cellular immunity. While these agents can be highly effective against rejection, their effects are broad and not donor antigen-specific. As a result, immunity against pathogens is also blunted, and infections are the leading cause of morbidity and mortality in transplantation patients [1].

A key mechanism of immune organ rejection is reactivity of host T cells against alloantigens within graft tissue. In normal T cell development, T cell receptors (TCRs) recognizing self-antigens are negatively selected in the thymus. Not having undergone this selective process against differing donor tissue antigens, host T cells react against transplanted donor cells, particularly non-self Major Histocompatibility Complex (MHC) molecules, so-named when discovered as the major determinants of tissue rejection [2]. During cell-mediated rejection, alloantigen-specific T cell infiltrates graft tissue [3–5]. The oligoclonality of these graft infiltrating lymphocytes (GILs) in human transplanted solid organs has been suggested by examining the relative frequencies of T cell receptor (TCR) beta chain variable (BV) gene families in T cell lines derived from both renal and cardiac graft tissue [6–9], as well as T cell populations isolated directly from renal graft tissue [10–13]. These studies circumstantially infer clonality in GIL populations by observing over-representation of certain TCR BV families, either through flow cytometric [6,11] or molecular genetic quantification of TCR BV gene distributions [7,8,10,12,13]. Persistence of this TCR BV skewing has been demonstrated over the course of clinical rejection [14,15], suggesting that the oligoclonal population of GILs comprise stable expanded clones mediating cellular rejection. However, there has been little direct human evidence that persistent skewing of BV family distributions reflects stability of clonal populations (TCR sequences) within those families. Datema *et al* [7] examined MHC class I-restricted T cell clones isolated from cultured GIL cell lines from endomyocardial biopsies of a transplanted heart in a single subject between days 8 and 129 after transplantation, but identified alloreactive T cell clones at only one time point.

Detailed characterization of the degree of clonal breadth and persistence of GILs in solid organ transplantation has the potential to provide important clinical insights about the diversity of T cells causing graft rejection, as well as designing more targeted therapies. In this study, we assess the graft-infiltrating CD8^+^ T cells following human hand transplantation, over a period of 178 days. Rather than long-term culture and cloning of T cells as in prior studies, we survey the bulk population of short-term expanded GILs to define the breadth and persistence of clonal expansions.

## Materials and Methods

### Study subject

A 27 year-old woman (HLA A2, A11, B35, B51, DR4, DR7, DQ2, and DQ8) was the recipient of a right hand from a male donor (HLA A1, B8, B72, DR8, DR17, DQ2, and DQ4). The human leukocyte antigen (HLA) profile was provided by the United Network for Organ Sharing (UNOS). This subject received the experimental hand transplantation under a research protocol approved by the UCLA Institutional Review Board, and provided written informed consent for participation. Detailed clinical data on this subject will be presented in a separate publication.

### Histopathological evaluation of rejection

Punch biopsies were obtained from the skin of the transplanted hand and evaluated by the UCLA Pathology Department. Assessment of the inflammatory cell infiltrate was used to assign a rejection grade based on standard Banff Criteria [16].

### CD8^+^ T lymphocyte expansion from GILs

To assess the cytotoxic T lymphocyte population in the transplanted tissue, bulk CD8^+^ T lymphocytes were selectively expanded from 3mm skin punch biopsies from the transplanted hand by culture of the tissue with 2 x 10^6^ autologous irradiated (3000 rad) PBMCs and a CD3:CD4 bispecific antibody that inhibits CD4^+^ T cells and stimulates CD8^+^ T cells [17], in a 24-well plate with RPMI 1640 (Sigma, USA) supplemented with 10% heat inactivated fetal calf serum, 10mM N-2-hydroxyethylpiperazine-N9-2-ethanesulfonic acid (HEPES), 2mM glutamine, 0.5ug/ml of pipercillin/tazobactam, 0.125ug/ml amphotericin B, and 50U/ml recombinant human interleukin-2 (NIH AIDS Reference and Reagent Repository). This bispecific antibody (available through the NIH AIDS Reference and Reagent Repository) was produced by fusing hybridomas producing anti-human CD3 and anti-human CD4, and purifying the heavy and light chain combination yielding antibodies with one arm recognizing each specificity (anti-CD3 heavy/light chain paired with anti-CD4 heavy/light chain). The cells were fed every 3–4 days and cultured for 14 days, and evaluated for percentage of CD8^+^ T cells by flow cytometry (Simultest, Becton Dickinson). For cultures that were <70% pure, additional isolation was performed using the EasySep Human CD8^+^ T Cell Enrichment Kit (STEMCELL Technologies, Inc., Canada) or the MACS CD8^+^ T Cell Isolation Kit (Miltenyl Biotec, Germany) selection.

Peripheral blood mononuclear cells (PBMCs) were isolated by Ficoll-Hypaque gradient from heparinized peripheral blood and expanded in parallel with CD3:CD4 bispecific antibody as previously described [18].

### T cell receptor (TCR) spectratyping

Variable gene families are named according to the international ImMunoGeneTics (IMGT) information system nomenclature. TCR alpha and beta chain spectratyping including quantitative PCR was performed as previously described [19]. Briefly, total RNA was isolated from CD8^+^ T cells (TRIzol reagent, Invitrogen, USA), and reverse transcribed to cDNA (High-Capacity Reverse Transcription Kit, Applied Biosystems, USA). Quantitative PCR (IQ5, BioRad, USA) was performed using alpha or beta variable (AV or BV) region-specific forward primers paired with alpha or beta constant (AC or BC) region-specific reverse primers. For amplification of BV families, quantitation was performed using a 5’-labeled Cy5 probe in the BC region, and the reverse primers were fluorescently tagged with fam, vic, or ned. BV family 6a was excluded from analysis due to primer cross-specificity to BV27. A plasmid containing the BV20 gene served as a quantitation standard. For amplification of AV families, quantitation was performed using a 5’-labeled Cy5 probe in the AC region, and the reverse primers were fluorescently tagged with fam, vic, ned, or pet. A plasmid containing the AV8 gene product served as a quantitation standard. The size distributions of amplified PCR products for each family were assayed by capillary electrophoresis (3130 Genetic Analyzer, Applied Biosystems, USA) and analyzed to determine the area under the curve of each peak (GeneMapper v3.7, Applied Biosystems, USA). The relative concentration of each family was calculated as the ratio of its copy number to the median copy number across all families. The relative concentration of each peak was calculated based on the fraction of the area under the curve of each peak within the total family.

### Genetic sequencing of TCR BV chains

When spectratyping revealed a single dominant expansion within a BV family, the PCR product for the whole family was analyzed for a dominant sequence by bulk sequencing as previously described [19]. When this did not reveal a single dominant sequence, or if a family contained more than one peak expansion, the PCR product was cloned by TOPO TA cloning (Invitrogen) followed by sequencing of at least 10 clones (selected as transformed bacterial colonies) from the family.

### Statistical analysis

Linear regression analysis was performed for comparison of AV and BV spectratype peak expansions. The Czekanowski Similarity Index was calculated for comparisons of BV spectratype profiles as % Similarity = 200 x (Σ_*n*_ minimum (p_a_, p_b_) ÷ Σ_*n*_ (p_a_ + p_b_)), where:

*n* corresponds to the peaks in the spectratype, and
p_a_ and p_b_ correspond to the sizes of the same peak for the two comparison samples a and b

## Results

### Clinical course after hand transplantation

**Fig 1:**
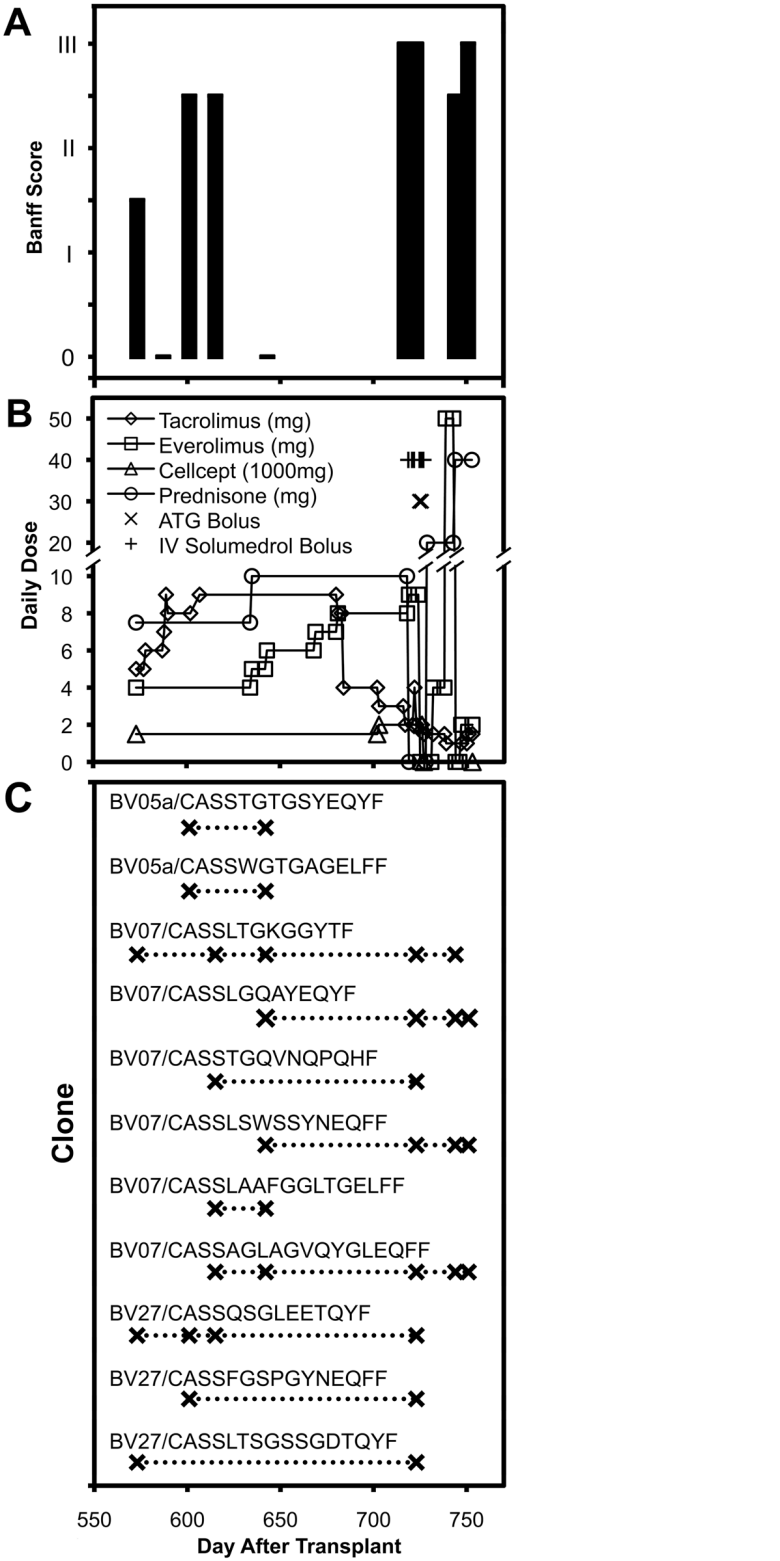
Rejection status, immunosuppressive drug regimen, and T cell receptor clonal persistence after hand transplantation. The histopathologic Banff score (A), immunosuppressive drug doses (B), and identified persistent T cell receptor clones listed in Table 1 (C) are plotted over time.

**Fig 2:**
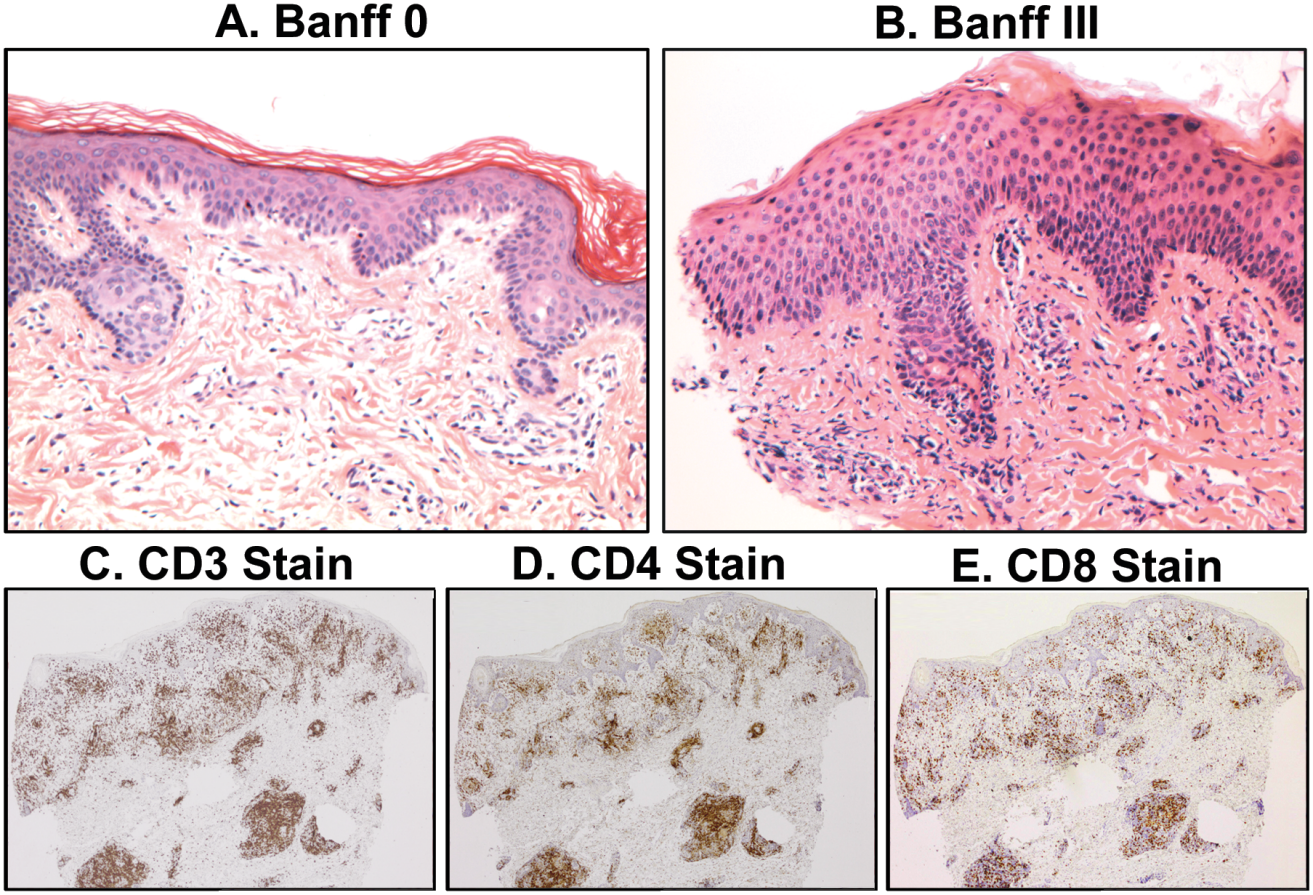
Histopathology of the transplanted hand. Examples of hematoxylin/eosin staining of biopsies from the transplanted hand are shown (20x). An increase in necrotic keratinocytes along the D-E junction (A) and epidermal spongiosis and lymphocyte exocytosis (B) seen with associated Banff criteria of 0 and III, respectively.

Serial skin biopsies were performed to evaluate for immune rejection. Prior to day 717 after transplantation, the patient had intermittent episodes of mild to moderate rejection with adjustments to the anti-rejection medication regimen (Figs 1 and 2). However on day 717 the subject presented with severe inflammation of the transplanted hand after nonadherence to medications, and was found to have severe rejection. She was treated with three high doses of solumedrol followed by anti-thymocyte globulin (ATG), but ultimately the patient elected to discontinue treatment and the transplanted hand was resected on day 771. The fully detailed clinical course will be reported elsewhere.

### Oligoclonal expansions of infiltrating CD8^+^ T cells were observed in the graft and not in the peripheral blood

**Fig 3:**
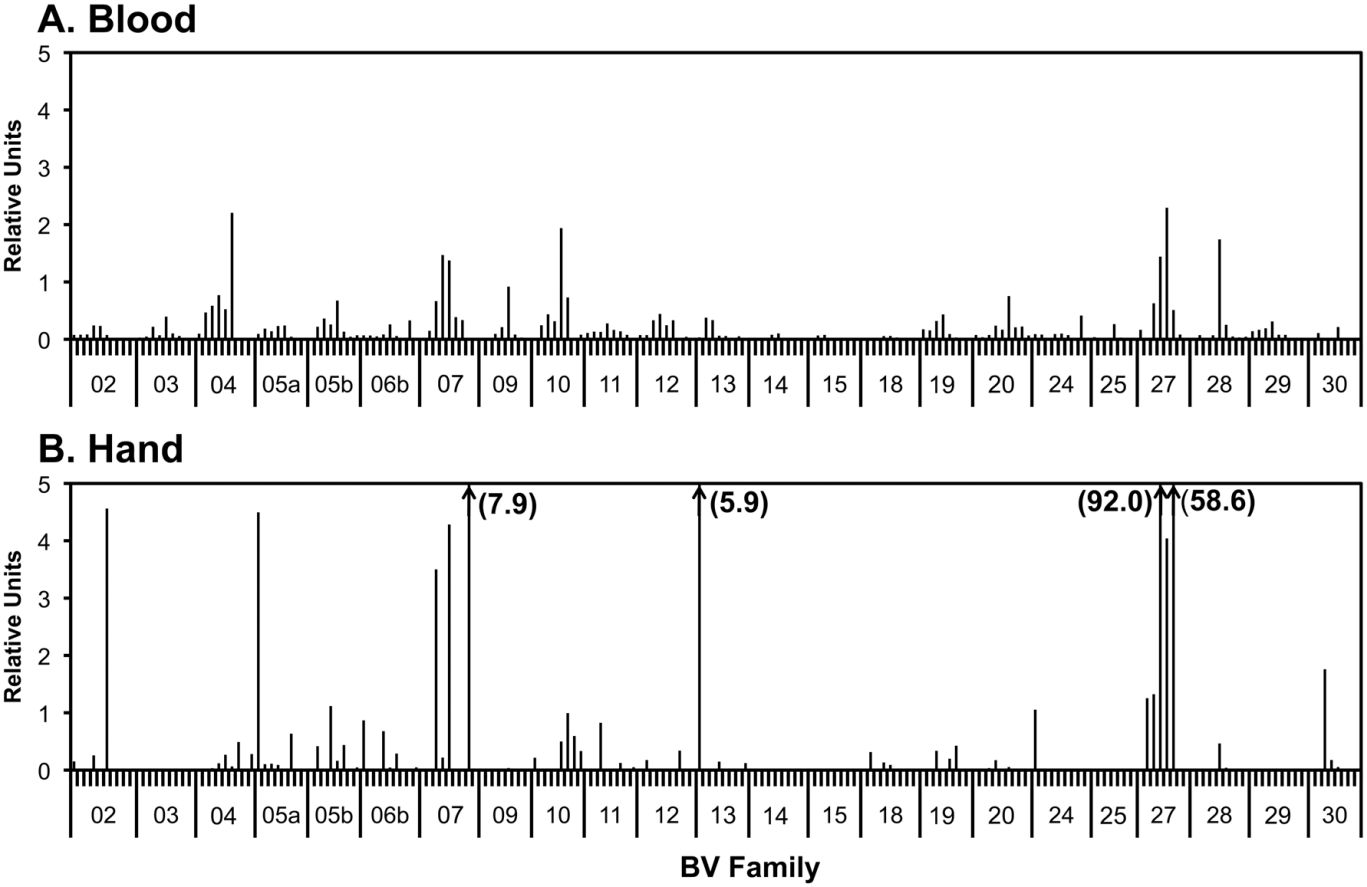
TCR BV spectratype profiles of CD8^+^ T cells expanded from peripheral blood and transplanted hand skin biopsy at day 573 after transplantation. CD8^+^ T cells were expanded from the blood (A) or biopsy (B) and harvested for RNA isolation and T cell receptor spectratyping as described in the Methods and Materials. “Relative units” refers to the ratio of each peak to the median concentration of all BV families.

Given their major immune effector role as cytolytic cells, CD8^+^ T lymphocytes were expanded from graft skin and examined for size distributions of TCR BV chains by a quantitative spectratyping technique incorporating real-time PCR [19]. On day 573 after transplantation (during an episode of moderate rejection, Banff II-III), the size distributions within the BV families in blood were relatively normally Gaussian, while those in the hand showed marked skewing due to oligoclonal expansions (Fig 3). Similar patterns were observed for CD8^+^ T cell AV size distributions in blood and hand (S1 Fig), indicating tissue compartmentalization of alloreactive T cells. Based on control spectratype profiles [19], expansions were defined as size peaks >2 units in magnitude.

### Longitudinal evaluation of graft-infiltrating CD8^+^ T cells demonstrated repeated skewed peaks over time

**Fig 4:**
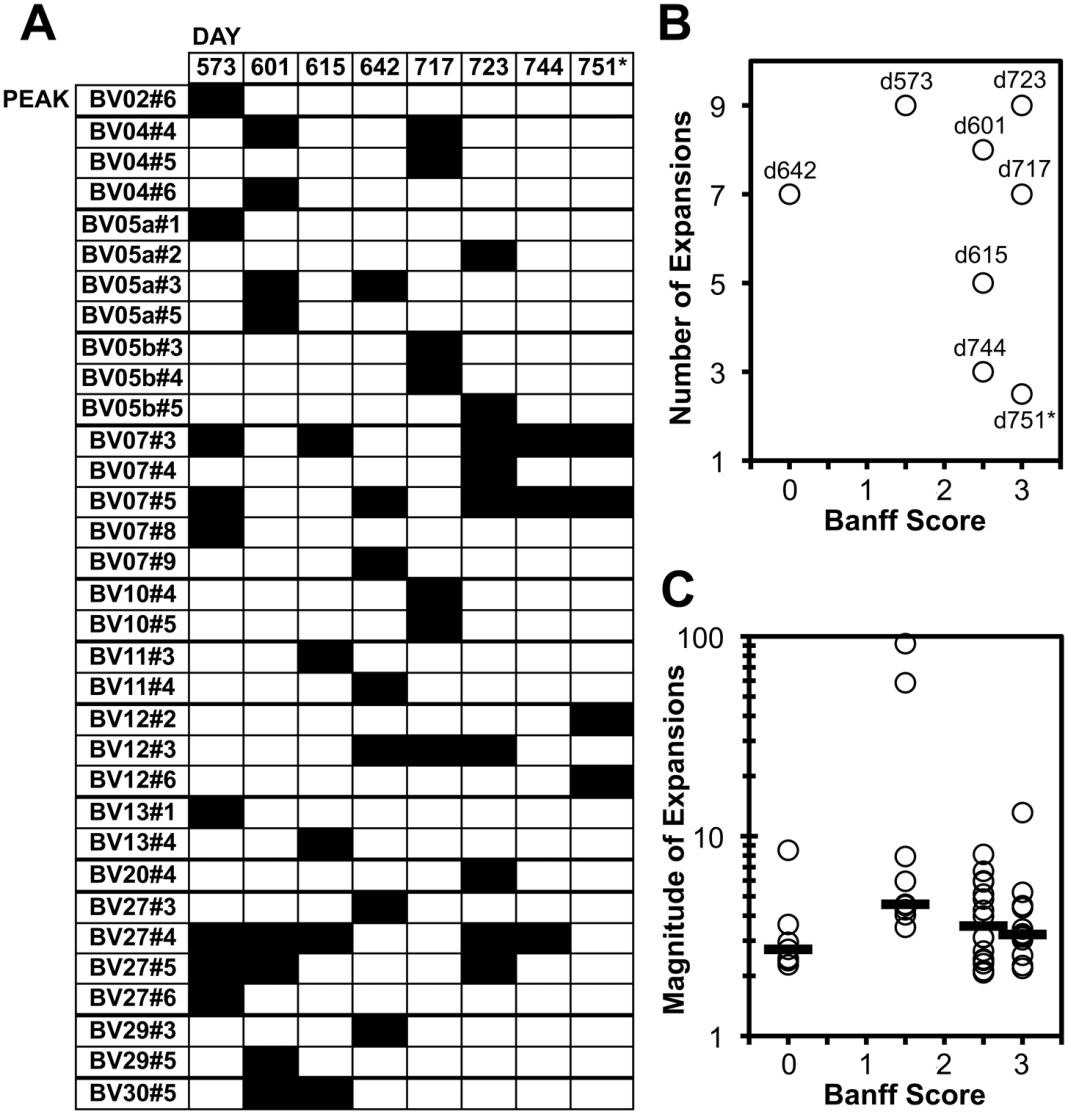
Spectratype BV peak expansions seen over time in CD8^+^ T cells from the transplanted hand. Spectratypes for BV families were performed (as in Fig 3) at the indicated time points. A. All expanded peaks (>2 relative units magnitude) are indicated by black shading, according to BV family and peak size. B. The number of observed expansions is plotted against the Banff score at each time point. C. The magnitudes of observed expansions are plotted against the Banff score at each time point. *The results for duplicate sampling on day 751 were combined for panel A, and averaged for panel C.

Transplanted hand skin biopsies were evaluated longitudinally in this manner between days 573 and 751. There were spectratype peak expansions seen in every tested time point, with a mean of 5.9 (±2.7) expansions per biopsy (Fig 4). Multiple expanded peaks were seen at more than one time point. The most repeated peak expansions were found in families BV07 and BV27, including two peak sizes (#3 and #5) in BV07 that were seen at five of seven time points each, and two peak sizes (#4 and #5) in BV27 that were seen in five and three of seven time points, respectively (Fig 4A). Comparing the numbers (Fig 4B) or magnitudes (Fig 4C) of expansions to the rejection status showed no clear relationship of clonal breadth or magnitude of infiltrating CD8^+^ T cells. The breadth of expansions was least at days 744 and 751, when there was severe rejection and likely vascular compromise just before resection of the hand, perhaps due to nonspecific influx of circulating lymphocytes. Similar results were seen for AV spectratyping (S2 Fig), although there were more expansions seen, perhaps due to the known incomplete allelic exclusion of AV chains [20] or better sensitivity of the AV PCR primers. Overall, these results suggested likely stability of clones within graft-infiltrating T cells, consistent with other studies in animal organ rejection models using similar spectratyping approaches [21,22].

### Sampling from transplanted hand skin biopsies yielded incomplete representation of the repertoire of infiltrating T cell clones

**Fig 5:**
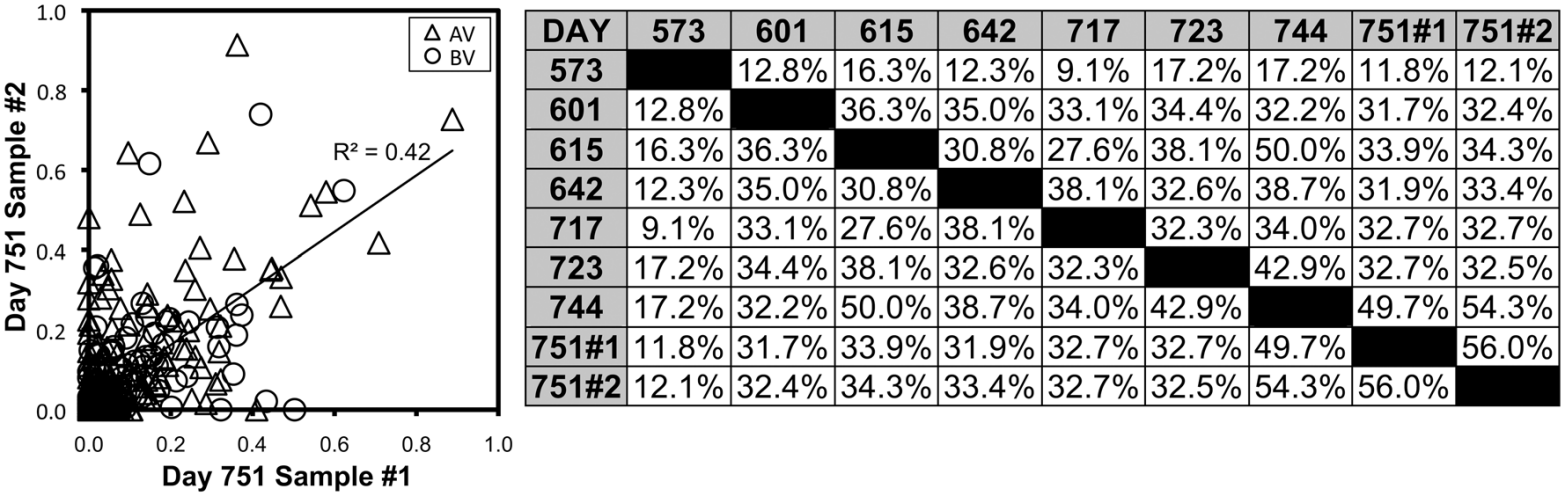
Correlation of CD8^+^ T cell spectratype profiles between duplicate samples and between time points. Left: All peaks in AV (triangles) and BV (circles) spectratypes from duplicate hand biopsies on day 751 after transplantation are plotted against each other (log_10_ scale for peak size +1). The *p* value was <0.001 for the correlation. Right: Comparisons of BV spectratype profiles (Czekanowski Similarity Index) are given for each time point and the duplicate samples on day 751.

To assess the adequacy of sampling for these analyses, two separate skin biopsies from day 751 after transplantation were processed in parallel. When all AV and BV peak sizes were compared between samples, there was a modest (r^2^ = 0.42) but significant (*p* <0.001) correlation of the two biopsies (Fig 5A). Across all 485 AV and BV spectratype peaks analyzed, 471 were negative in both samples (peak height <2), 4 were positive in both samples, 2 were positive in the first sample but negative in the second sample, and 8 were positive in the second sample but negative in the first. These results indicate that the 3mm skin biopsies were inadequate to represent the entire population graft-infiltrating T cells, and thus that our evaluations under-sample the populations of T cell clonal expansions in the graft.

### Despite sampling limitations, specific CD8^+^ T lymphocyte clonal expansions were seen to persist over time

Despite the under-sampling indicated by comparing the duplicate biopsies on day 751 (Fig 5A), there was similarity between spectratype profiles across time (Fig 5B) that suggested stability over time. To evaluate this in more detail, each BV family with at least one recurrent expansion (Fig 4) was evaluated for TCR BV sequences. If the family contained a single expanded peak, the spectratyping PCR product was subjected to bulk sequencing to reveal if the peak reflected a single dominant clone in that family. For families with more than one expanded peak, or when bulk sequencing did not identify a single dominant sequence, molecular cloning of the PCR product was performed to obtain at least 10 BV sequences per family. This process yielded 11 TCR BV clonal sequences that were present at multiple time points (Table 1), as well as 95 sequences that were seen only at single time points (not shown). The repeated sequences included two in BV05, six in BV07, and three in BV27. The most repeated peak expansions in BV07 (#3 and #5) and BV27 (#4 and #5) all had corresponding sequences identified, and there were two cases where single peak expansions corresponded to more than one sequence (BV05a #3, BV07 #3). There was identification of one repeated sequence that was not seen at any time point as a peak expansion by spectratyping (BV07 #7), perhaps obscured by the other expansions present. Eight sequence clones were found to persist at least 100 days, the longest being at least 171 days during the 178 days of surveillance. Several were observed to be present on day 642, despite minimal rejection at that time (Banff 0). Overall, these results indicate clear evidence of long-lived CD8^+^ T cell clones infiltrating the transplanted hand, despite the sampling limitations posed by isolation of T cells from skin biopsies.

**Table 1:** Persistent TCR clones infiltrating the transplanted hand. Any BV family demonstrating a peak expansion at more than one time point was subjected to bulk sequencing of the uncloned PCR product for the whole family. If no clearly dominant sequence was obtained, clonal sequencing (TOPO-TA) of at least 12 clones was performed for that family. For each listed peak and detected CDR3 sequence corresponding to that peak size, whether that sequence was the dominant sequence seen in bulk sequencing or the frequency of that sequence within clonal sequences is given for the indicated times after transplantation. The final column gives the minimum duration of each clonal sequence (time between first and last detection).

	Day After Transplantation									
Expanded Peak/CDR3 Sequence	573	601	615	642	717	723	744	751*	751**	Min. Duration
**BV05a#3/CASSWGTGAGELFF**	<Dom	10/17	ND	1/10	ND	<Dom	ND	ND	ND	**41 Days**
**BV05a#3/CASSTGTGSYEQYF**	<Dom	1/17	ND	6/10	ND	<Dom	ND	ND	ND	**41 Days**
**BV07#3/CASSLTGKGGYTF**	4/23	ND	2/14	4/25	ND	1/10	1/26	0/21	0/14	**171 Days**
**BV07#3/CASSLGQAYEQYF**	0/23	ND	0/14	1/25	ND	1/10	5/26	6/21	2/14	**109 Days**
**BV07#4/CASSTGQVNQPQHF**	0/23	ND	1/14	0/25	ND	2/10	0/26	0/21	0/14	**108 Days**
**BV07#5/CASSLSWSSYNEQFF**	0/23	ND	0/14	6/25	ND	2/10	8/26	1/21	1/14	**109 Days**
**BV07#7/CASSLAAFGGLTGELFF**	0/23	ND	1/14	1/25	ND	0/10	0/26	0/21	0/14	**27 Days**
**BV07#8/CASSAGLAGVQYGLEQFF**	0/23	ND	1/14	1/25	ND	2/10	2/26	3/21	0/14	**136 Days**
**BV27#4/CASSQSGLEETQYF**	12/16	5/12	>Dom	<Dom	ND	10/22	<Dom	ND	ND	**150 Days**
**BV27#5/CASSFGSPGYNEQFF**	0/16	1/12	<Dom	<Dom	ND	5/22	<Dom	ND	ND	**122 Days**
**BV27#6/CASSLTSGSSGDTQYF**	3/16	0/12	<Dom	<Dom	ND	1/22	<Dom	ND	ND	**150 Days**

“<Dom” Indicates that the specified sequence was not the dominant sequence revealed by bulk sequencing of the whole family

“>Dom” Indicates that the specified sequence was the dominant sequence revealed by bulk sequencing of the whole family

“ND” Not Done

*Obtained from first biopsy on Day 751

**Obtained from duplicate biopsy on Day 751

## Discussion

Our data provide a comprehensive evaluation of cellular immune rejection of a transplanted human hand over the course of 178 days. Although this is a single case, to our knowledge this is the most comprehensive longitudinal assessment of TCR clonal sequences in a human transplanted solid organ. In contrast, most prior studies of this topic have evaluated only concentrations of whole BV families. An oligoclonal population of GILs in the skin was detectable throughout the clinical course, even when clinical rejection appeared minimal, and despite immunosuppressive therapy, while peripheral blood TCR size distributions were relatively normal. These results agree with limited human data regarding GIL BV restriction during rejection [6–14] in the setting of a relatively unperturbed peripheral blood T cell population [23].

While both CD4^+^ and CD8^+^ T lymphocyte responses are involved in rejection [24], we focused on the CD8^+^ T lymphocytes, as these are generally the front line effector cells mediating cellular damage through cytolysis. Although the CD4^+^ T cell responses can also produce cellular rejection through either direct MHC II molecule allorecognition [25] or the indirect presentation of minor histocompatibility antigens [26], biopsy sample size constraints did not allow for characterization of both these populations.

Our analysis showed that three millimeter diameter punch biopsies of transplanted hand skin are inadequate to provide full sampling of CD8^+^ T cells infiltrating the graft, and thus our analyses likely underestimate the degree of clonal persistence. Given that each 3mm diameter biopsy (7.1mm^2^) revealed an average of 5.9 clones, if it is assumed that the 26 repeated BV spectratype expansions (Fig 4A) were the total population, even 4 biopsies would be insufficient to cover the total population. This limitation also makes it difficult to correlate our data quantitatively (e.g. numbers expanded clones and the magnitudes of expansions) to the degree of rejection, or to accurately assess the frequencies of clones over time. Finally, the need for short term culture of the cells also introduces potential bias in our findings, although two studies using this method have demonstrated that this expansion protocol causes minimal bias in the detection of antigen-specific T cells [18,27]. Despite these limitations, there were clones that were shown to persist longitudinally, suggesting relative stability of the T cell clones causing rejection. This relative clonal stability across different levels of rejection was consistent the results of a recent study of transplanted skin rejection, which suggested that the inflammatory profile (and therefore immune cell phenotype) is a key determinant of rejection status [28].

With these above caveats, no correlation between rejection status and clonal breadth of GILs was observed. It is unclear whether this was due to sampling limitation or a true lack of a correlation due to the underlying immunology or changing anti-rejection treatments. It is notable that the lowest degree of clonal breadth was observed at the end of the clinical course when rejection was most severe (S3 Fig); at that point there was severe tissue breakdown with inflammation, and thus the reduced oligoclonality may reflect nonspecific influx of circulating T cells.

The BV usage of clones in the transplanted hand appears biased towards certain families, within our sampling limitations. The 11 clones identified to persist over time fell in only three BV gene families of the 23 analyzed, and six of the 11 were in the BV07 family. Because the three families with these expansions are among the largest, it is thus unclear whether this was true bias or just proportional distribution based on magnitude of different BV families. However, in BV05a and BV07, there were multiple clones within single peak expansions, which would seem unlikely to be due to random chance given the nearly 200 peaks assessed in the BV spectratype. BV and clonotype bias tends to be reproducible and predictable in defined animal transplant models, determined by the particular genetic pairing [22,29]. In this case, it is unclear what human genetic mismatches may have determined the observed BV bias.

Understanding and predicting BV usage bias in graft rejection could be clinically useful. Prediction of BV usage useful for therapeutic specificity by targeting only those BV families involved in rejection for deletion [14,30], rather than nonspecifically via agents such as OKT3. In genetically controlled animal transplant models, BV usage of alloreactive T cells is predictable [29,31,32] and stable over time [22,33]. As proof-of-concept, administration of specific BV-depleting antibodies in mice has been shown to prolong graft survival [34]. Given the polymorphic and diverse nature of HLAs, the genetic interactions between recipient T cells and donor HLA molecules may be difficult to predict, although there are situations where individuals of a certain HLA type tend to have shared “public” TCR usage against specific pathogens [35]. Certain host/donor HLA combinations may yield predictable BV dominance in rejection, allowing a BV-depleting approach to increase graft longevity. The biased BV usage observed in our subject raises the question of a possible association between combinations of HLA mismatches among the donor and recipient; further studies of large numbers of subjects may reveal such patterns. Although this is a study of a single person, to our knowledge this is the most comprehensive T cell receptor analysis of any human organ transplantation. Future studies in other transplanted organs will be important to determine the generality of our findings and the potential to predict BV usage for targeted T cell ablation to prevent or treat rejection.

In summary, we have demonstrated the presence of an oligoclonal population of CD8^+^ T cells infiltrating a transplanted hand in the setting of ongoing immunosuppressive therapy. Despite sampling limitations, individual CD8^+^ T lymphocyte clones were observed to persist across multiple time points through varying degrees of cellular rejection. A more complete understanding of these processes will allow for the design of specific interventions for the prevention and treatment of cell-mediated rejection of transplanted organs.

## Supporting Information

S1 Fig CD8^+^ TCR AV spectratype profiles of blood and skin from the transplanted hand on day 573 after transplantation. Spectratyping of TCR AV genes of CD8^+^ T lymphocytes from blood (A) and skin from the transplanted hand (B) was performed as described in the Methods and Materials.
(TIF)

S2 Fig Spectratype AV peak expansions seen over time in CD8^+^ T cells from the transplanted hand. Spectratypes for AV families were performed (as in Fig 3) at the indicated time points. A. All expanded peaks (>2 relative units magnitude) are indicated by black shading, according to AV family and peak size. B. The number of observed expansions is plotted against the Banff score at each time point. C. The magnitudes of observed expansions are plotted against the Banff score at each time point.
(TIF)

S3 Fig Spectratype AV and BV peak expansions over time. The numbers of AV and BV expansions (>2 relative units magnitude) are plotted for each tested time point. The numbers for day 751 are averaged for the two assays performed that day.
(TIF)
