# Correction: Clonal CD8^+^ T Cell Persistence and Variable Gene Usage Bias in a Human Transplanted Hand

**DOI:** 10.1371/journal.pone.0146008

**Published:** 2015-12-23

**Authors:** Joseph Y. Kim, Arumugam Balamurugan, Kodi Azari, Christian Hofmann, Hwee L. Ng, Elaine F. Reed, Suzanne McDiarmid, Otto O. Yang

There is an error in the first sentence under the subheading Study Subject, in the Materials and Methods section. The correct sentence is: A 27 year-old woman (HLA A1, B8, B72, DR8, DR17, DQ2, and DQ4) was the recipient of a right hand from a male donor (HLA A2, A11, B35, B51, DR4, DR7, DQ2, and DQ8).
