# Supplementary figures and images for: Clonal CD8+ T Cell Persistence and Variable Gene Usage Bias in a Human Transplanted Hand
Source: PLoS One. 2015 Aug 19;10(8):e0136235. doi: 10.1371/journal.pone.0136235 (PMC4546120; doi:10.1371/journal.pone.0136235)

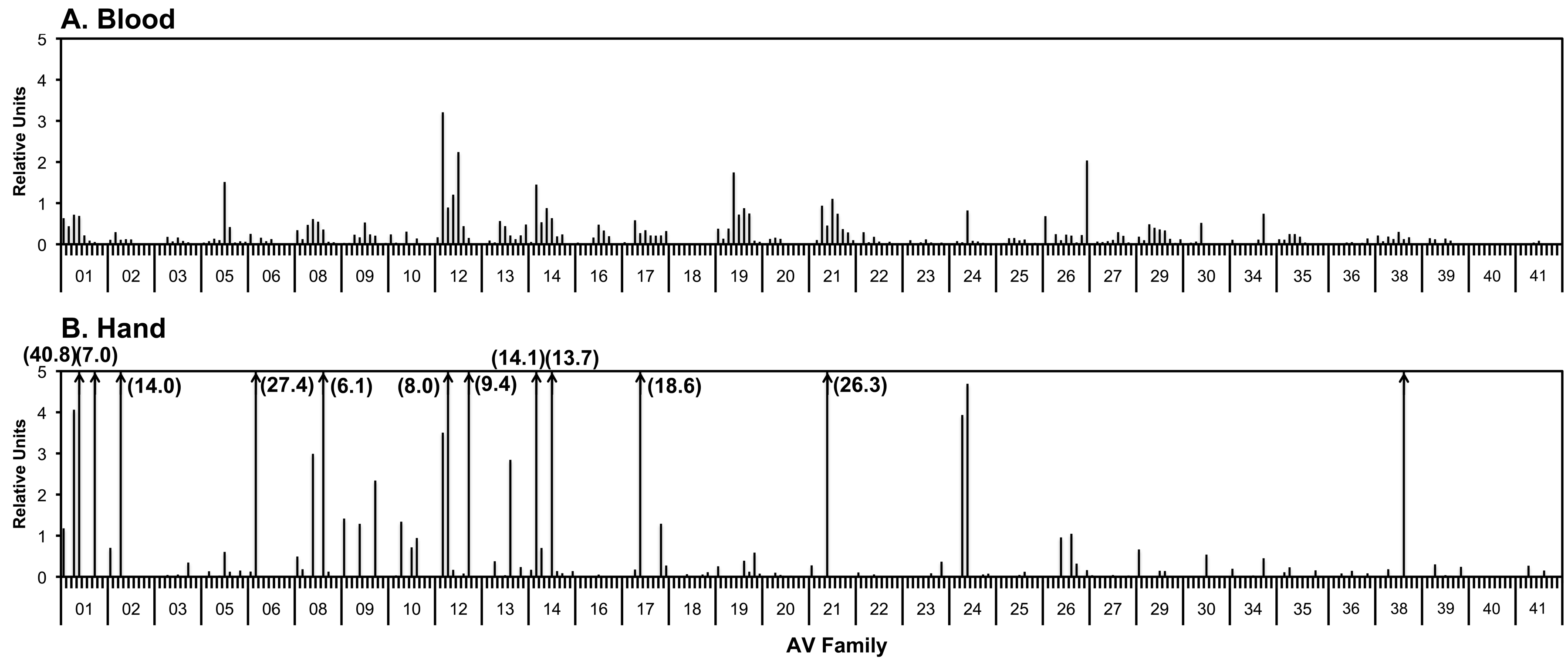

Supplement: S1 Fig — Spectratyping of TCR AV genes of CD8+ T lymphocytes from blood (A) and skin from the transplanted hand (B) was performed as described in the Methods and Materials. (TIF) [file pone.0136235.s001.tif]

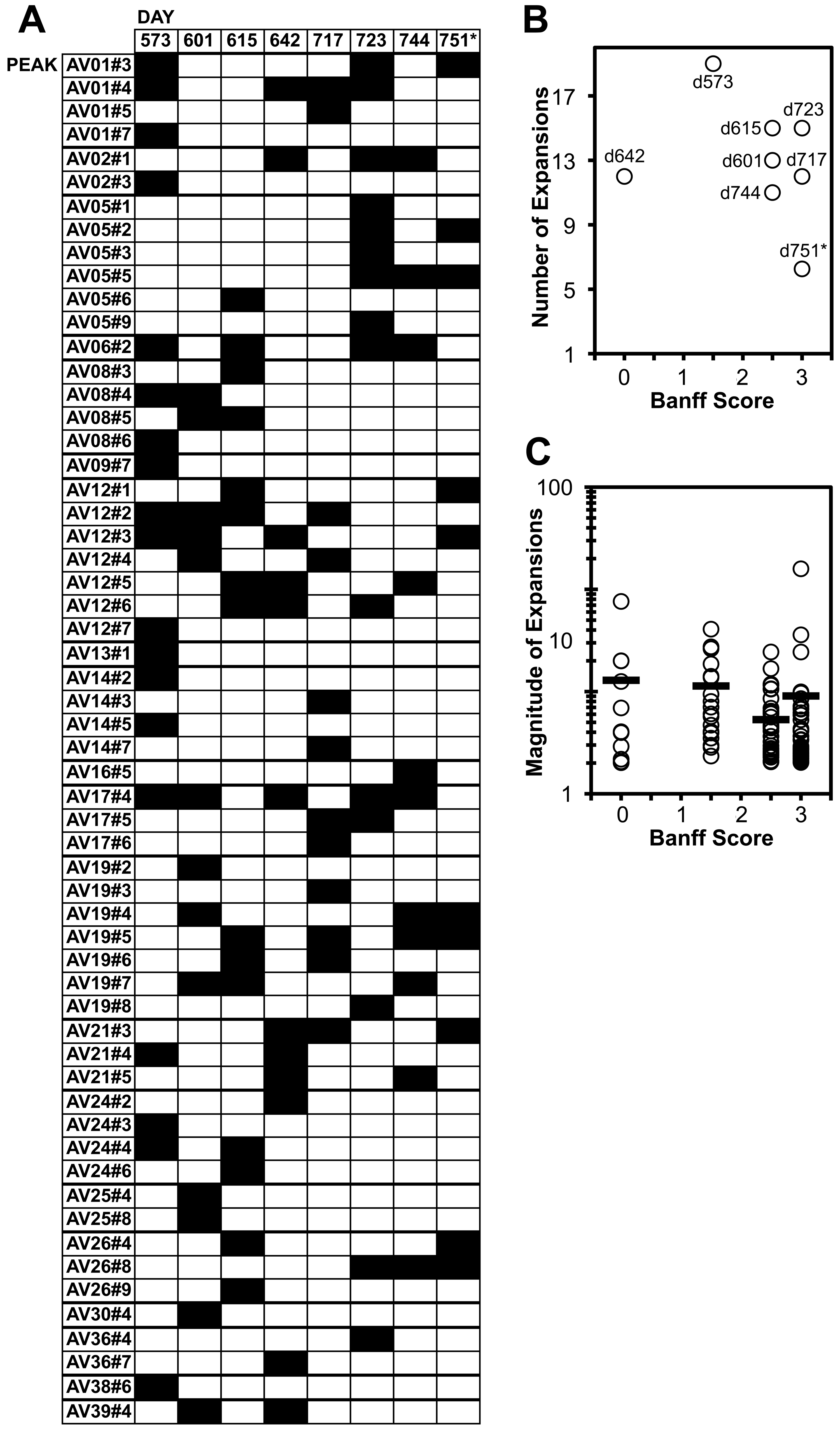

Supplement: S2 Fig — Spectratypes for AV families were performed (as in Fig 3) at the indicated time points. A. All expanded peaks (>2 relative units magnitude) are indicated by black shading, according to AV family and peak size. B. The number of observed expansions is plotted against the Banff score at each time point. C. The magnitudes of observed expansions are plotted against the Banff score at each time point. (TIF) [file pone.0136235.s002.tif]

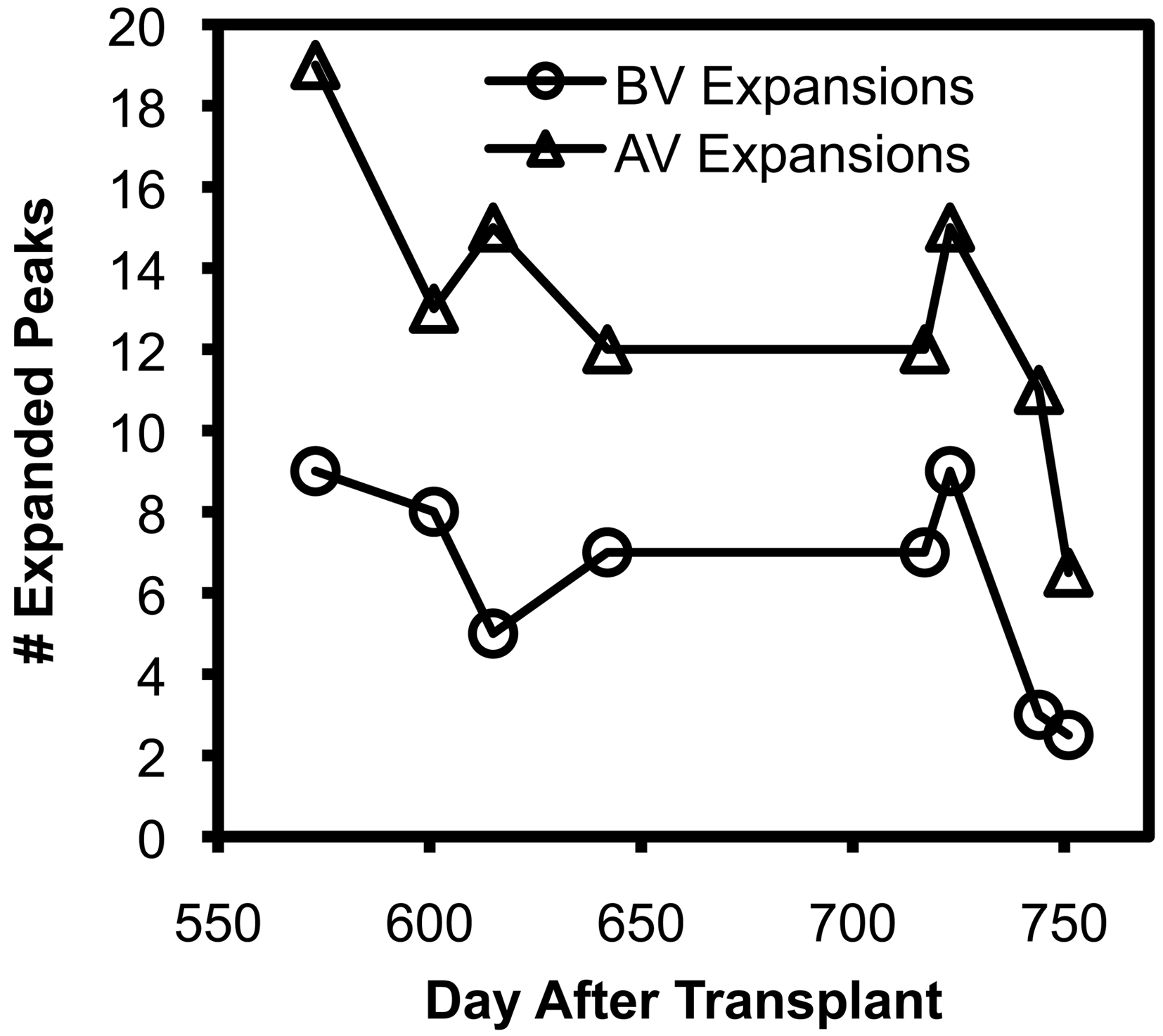

Supplement: S3 Fig — The numbers of AV and BV expansions (>2 relative units magnitude) are plotted for each tested time point. The numbers for day 751 are averaged for the two assays performed that day. (TIF) [file pone.0136235.s003.tif]
